# Micronutrient Intakes and Incidence of Chronic Kidney Disease in Adults: Tehran Lipid and Glucose Study

**DOI:** 10.3390/nu8040217

**Published:** 2016-04-20

**Authors:** Hossein Farhadnejad, Golaleh Asghari, Parvin Mirmiran, Emad Yuzbashian, Fereidoun Azizi

**Affiliations:** 1 Nutrition and Endocrine Research Center, Research Institute for Endocrine Sciences, Shahid Beheshti University of Medical Sciences, Tehran 19395-4763, Iran; hosein.farhadnejad@gmail.com (H.F.); g_asghari@hotmail.com (G.A.); e_yuzbashian@yahoo.com (E.Y.); 2 Department of Clinical Nutrition and Dietetics, National Nutrition and Food Technology Research Institute, Faculty of Nutrition Sciences and Food Technology, Shahid Beheshti University of Medical Sciences, Tehran 19395-4741, Iran; 3 Endocrine Research Center, Research Institute for Endocrine Sciences, Shahid Beheshti University of Medical Sciences, Tehran 19395-4763, Iran; azizi@endocrine.ac.ir

**Keywords:** chronic kidney disease, micronutrients, vitamins, minerals

## Abstract

The aim of this study was to investigate the associations between micronutrient intakes and the 3.6-year incidence of chronic kidney disease (CKD) in adults. This cohort study was conducted, within the framework of the Tehran Lipid and Glucose Study, on 1692 subjects, aged ≥30 years, without CKD at the baseline. Dietary intakes were collected using a valid and reliable food-frequency questionnaire. Anthropometrics and biochemical measurements were taken. Chronic kidney disease was defined as eGFR < 60 mL/min/1.73 m^2^. The mean age of participants was 43.3 ± 11.4 years. In the fully adjusted model, individuals in the top quintile of folate (OR: 0.44, 95% CI: 0.24–0.80), cobalamin (OR: 0.57, 95% CI: 0.34–0.93), vitamin C (OR: 0.38, 95% CI: 0.21–0.69), vitamin E (OR: 0.45, 95% CI: 0.22–0.92), vitamin D (OR: 0.39, 95% CI: 0.21–0.70), potassium (OR: 0.47, 95% CI: 0.23–0.97) and magnesium (OR: 0.41, 95% CI: 0.22–0.76) had decreased risk of CKD, and in the top quintile of sodium (OR: 1.64, 95% CI: 1.03–2.61), subjects had increased risk of CKD, in comparison to the bottom quintile. No significant associations were found between the intakes of other micronutrients. High intake of several micronutrients including vitamins C, E, D, cobalamin, folate, magnesium, and potassium was associated with a decreased risk, while sodium was associated with an increased risk of incident CKD.

## 1. Introduction

Chronic kidney disease (CKD) is a progressive renal impairment accompanied by structural and functional damage. Pathological factors including renal failure markers (such as abnormal xerographic, albuminuria, and increased urinary sodium) or glomerular filtration rate (GFR) lower than 60 mL/min/1.73 m^2^ persistent for over three months are among the main characteristics of CKD [1,2]. The incidence and prevalence of CKD are fast increasing worldwide [3]. In a study, the overall prevalence of CKD was 11% in an Iranian population, aged over 20 years [4]. Cardiovascular disease, anemia, mineral and bone disorders, peripheral neuropathy, cognitive dysfunction, and increased infection are the important complications of CKD [5]. Of the several risk factors of CKD, including diabetes, hypertension, obesity, sedentary lifestyle, alcohol consumption [1,6], diet is a modifiable environmental risk factor, which plays an important role in the incidence or development of CKD [7,8,9].

Some studies have indicated that the Dietary Approaches to Stop Hypertension (DASH) and the Mediterranean dietary patterns which are rich in protective nutrients such as antioxidant vitamins (e.g., vitamin E, C, A), potassium, magnesium, calcium, fiber, ω-3 fatty acids, and phytochemicals, can affect kidney function and decrease the risk of CKD [7,8]. Despite several studies available on effect of micronutrients on the risk factors of CKD, including obesity, diabetes, and hypertension, limited data are available regarding the association between micronutrient intakes and risk of incident CKD. To the best of our knowledge, evidence on the association between micronutrient intakes and risk of CKD is limited to cross-sectional studies [10,11,12], indicating controversial findings for sodium and phosphorus, and protective effects of potassium, magnesium, calcium, and folate on the prevalence of CKD. These controversial findings may be explained by cross-sectional design of studies, different dietary assessment tools, and different adjustment of potential confounding factors.

Because limited data are available regarding the association of micronutrient intakes with the risk of CKD, the association between most of the dietary micronutrients and risk of incident CKD remains unclear. The aim of the present study was to examine the relationship between the risk of incident CKD and intakes of dietary vitamins (thiamin, riboflavin, niacin, pyridoxine, folate, cobalamin, vitamins A, D, E, and K) and minerals (sodium, potassium, calcium, magnesium, phosphorus, selenium, and zinc) after 3.6 years of follow-up, using data from the Tehran Lipid and Glucose Study (TLGS).

## 2. Materials and Methods

### 2.1. Subjects

The current study, conducted within the framework of the TLGS, aimed at preventing non-communicable diseases (NCD) by developing programs promoting healthy lifestyles and reducing NCD risk factors [13]. Baseline data were collected from 15,005 participants, aged ≥3 years, residents under the coverage of three medical health centers in District No. 13 of Tehran, the capital city of Iran. Participants were followed up every 3 years to update their data on demographics and lifestyle, biochemical, clinical, and dietary measurements; the baseline survey was a cross-sectional study conducted from 1999 to 2001, and surveys 2 (2002–2005), 3 (2006–2008), and 4 (2009–2011) were prospective follow-up surveys.

In the third survey of the TLGS (2006–2008), from among 12,523 examined participants, 3462 were randomly selected for dietary assessment; of these, 2417 individuals, aged ≥30 years, were included in the current study. Of these, participants with missing data on covariates (*n* = 8), those with over- and under-reporting in dietary data (daily energy intake outside the range of 800–4200 kcal/day), (*n* = 59), and those with history of cancer or specific diet were excluded (*n* = 7). We also excluded individuals with baseline CKD (*n* = 390). Some individuals fell into more than one exclusion category. Participants were followed up to survey 4, for an average of 3.6 years. After exclusion of subjects with missing data on follow-up assessments (*n* = 302), 1692 participants remained for the final analysis (follow-up rate: 84%).

The protocol of this study was approved by the institutional ethics committee of the Research Institute for Endocrine Sciences, affiliated to the Shahid Beheshti University of Medical Sciences, Tehran, Iran. Written informed consent was obtained from all participants included in the study.

### 2.2. Dietary Intake Assessment

Dietary data were collected by using a valid, reliable 168-item semi-quantitative food frequency questionnaire (FFQ) [14,15,16]. Trained dietitians asked participants to designate their consumption frequency for each food item during the previous year on a daily, weekly, or monthly basis. Portion sizes of consumed foods, reported in household measures, were converted to grams.

Because the Iranian food composition table was incomplete, the USDA food composition table (FCT) was used as an alternative to calculate energy and nutrient intakes. The Iranian FCT was used for national foods not listed in the USDA FCT. Validity and reliability of the FFQ for nutrients were acceptable; the correlation coefficients between the FFQ and multiple 24 recalls were 0.53 and 0.39, and those between the two FFQs were 0.59 and 0.60 in male and female participants, respectively [15].

In the current study, vitamin and mineral content of foods including thiamin, riboflavin, niacin, pyridoxine, folate, cobalamin, vitamin A, vitamin D, vitamin E, vitamin K, sodium, potassium, calcium, magnesium, phosphorus, selenium, and zinc were calculated and divided into quintiles. Also, % Adequate Intake (AI) for sodium and potassium intakes and % of Estimated Average Recommended (EAR) of vitamin and other mineral intakes in subjects was estimated according to quintiles.

### 2.3. Physical Activity Assessment

To estimate physical activity level, the Modifiable Activity Questionnaire (MAQ) was used, and metabolic equivalent task (MET) minutes per week were calculated for all subjects [17]. This questionnaire has previously been modified and validated among young Iranians [18]. Light level of physical activity was considered MET < 600 min/week.

### 2.4. Clinical and Biological Measurements

Face-to-face private interviews were conducted by trained interviewers for completion of the pretested questionnaires. Essential information including age, smoking habits, and medication use was obtained. Height, weight, and waist circumference were measured, and body mass index (BMI) was calculated. Weight was recorded while the subjects were minimally clothed using digital scales and recorded to the nearest 100 g. Shoes and socks were removed before measurements were taken. Height was measured in a standing position, without shoes, using a stadiometer, while the shoulders were in a normal position. Body mass index (BMI) was computed as weight in kilograms, divided by height in meters squared. Waist circumference was measured at the level of the umbilicus, over light clothing, applying an unstretched shape tape meter, without any pressure to body surface; measurements were recorded to the nearest 0.1 cm. Systolic and diastolic blood pressure were measured twice in a sitting position after a resting period of 15 min, using a standard mercury sphygmomanometer, and the mean of two separate measurements was considered as subject’s blood pressure.

Blood samples were drawn between 7:00 a.m. and 9:00 a.m. from all study participants after 12–14 h overnight fasting; all blood analyses were done at the TLGS research laboratory on the day of blood collection. The analysis of samples was performed using Selectra 2 auto-analyzer (Vital Scientific, Spankeren, The Netherlands). Fasting plasma glucose (FPG) was measured using an enzymatic colorimetric method with glucose oxidase. Inter- and intra-assay CV were both 2.2% for FPG. For measurement of TGs, we used an enzymatic calorimetric method with glycerol phosphate oxidase. Inter- and intra-assay coefficients of variations (CV) for TGs were 0.6% and 1.6%, respectively. Total cholesterol (TC) was assessed with cholesterol esterase and cholesterol oxidase using the enzymatic colorimetric method. After precipitation of Apo lipoprotein β with phosphotungstic acid, HDL-C was measured. Inter- and intra-assay CV for both TC and HDL-C were 0.5% and 2%, respectively. Low density lipoprotein cholesterol (LDL-C) was calculated from the serum and TC, TG and HDL-C concentrations expressed in mg/dL using the Friedewald formula. Serum creatinine (cr) was measured according to the standard colorimetric Jaffe_Kinetic reaction method. Both intra-assay and inter-assay CVs were less than 3.1%. These analyses were performed using commercial kits (Pars Azmoon Inc., Tehran, Iran).

### 2.5. Definitions

Hypertension was defined as any of the following: Systolic blood pressure (SBP) ≥140, diastolic blood pressure (DBP) ≥90, or current use of antihypertensive drugs [19].

Diabetes was defined according to the criteria of the American Diabetes Association as fasting plasma glucose ≥126 mg/dL or 2 h post 75 g glucose load ≥200 mg/dL or current therapy for a definite diagnosis of diabetes [20].

We used the Modification of Diet in Renal Disease (MDRD) equation formula to express eGFR in mL/min/1.73 m^2^ of body surface area [21]. The abbreviated MDRD study equation is as follows:

eGFR = 186 × (Serum creatinine)^−1.154^ × (Age)^−0.203^ × (0.742 if female) × (1.210 if African-American) (1)

Patients were classified based on their eGFR levels by the national kidney foundation guidelines [2], as eGFR ≥ 60 mL/min/1.73 m^2^ as non-CKD and eGFR < 60 mL/min/1.73 m^2^ as CKD.

### 2.6. Statistical Analysis

Statistical Package for Social Sciences (version 15.0; SPSS Inc., Chicago, IL, USA) was used for data analyses and *p* values < 0.05 were considered statistically significant. The normality of all variables distribution was evaluated by histogram charts and Kolmogorov-Smirnov analysis. Normally distributed continuous variables are expressed as the mean ± SD and skewed continuous variables are reported as median (25–75 inter-quartile range). To compare quantitative variables between CKD and non-CKD groups, independent *t*-test and Mann-Whitney test were used for means and medians, respectively. Qualitative variables are reported as percentage and Pearson Chi-Square test was applied for comparison of variables between CKD and non-CKD groups. The association between micronutrient quintiles and incident CKD was investigated by multiple logistic regression, and odds ratio (OR) with 95% confidence intervals (CI) were reported. Age, physical activity, smoking, sex, energy intake, TGs, TC, BMI, hypertension, and diabetes were adjusted in models.

## 3. Results

Of 1692 subjects, 173 (10.2%) cases of CKD occurred after 3.6 years of follow-up. Mean (SD) age and BMI for participants were 43.3 (11.4) years and 27.6 (4.6) kg/m^2^, respectively at baseline.

Clinical and biochemical characteristics and energy intake of participants are reported in Table 1. Compared to non-CKD subjects, those with CKD were more likely to be female, older, less active, less smoker, hypertensive, and diabetic, have higher BMI, serum concentrations of TG and TC, HDL-C, and LDL-C; however, FPG did not differ between the two groups.

Multivariable-adjusted ORs for CKD across quintiles of vitamin intakes are given in Table 2. Of water soluble vitamins, higher intakes of folate (258.8% of EAR), cobalamin (369.7% of EAR), and vitamin C (445.4% of EAR) were significantly associated with lower risk of incident CKD. Comparing subjects in the highest to those in the lowest quintile of intakes, the multivariable ORs (95% CI) after adjusting baseline age, sex, physical activity, energy intake, smoking, TGs, TC, BMI, hypertension, and diabetes were 0.44 (0.24–0.80) for folate, 0.57 (0.34–0.93) for cobalamin, and 0.38 (0.21–0.69) for vitamin C. Also, of fat soluble vitamins, higher intake of vitamins E (163.3% of EAR) and D (40.9% of EAR) were significantly associated with decreased risk of incident CKD. Comparing subjects in the highest to those in the lowest quintile of intakes, the ORs (95% CI) in the fully adjusted models were 0.45 (0.22–0.92) for vitamins E and 0.39 (0.21–0.70) for vitamin D. A significantly decreasing linear trend of dietary folate, cobalamin, and vitamins C, E, and D for the risk of incident CKD was observed (*p* for trend < 0.05).

Of minerals, higher intakes of potassium (131.6% AI) and magnesium (195.7% EAR) were significantly associated with lower risk of incident CKD. Subjects with highest intakes of potassium (OR: 0.47, 95% CI: 0.23–0.97) and magnesium (OR: 0.41, 95% CI: 0.22–0.76) had lower odds of incident CKD, compared to those with the lowest intake after adjusting for age, sex, physical activity, smoking, energy intake, TGs, TC, BMI, hypertension, and diabetes; however, subjects in the highest quintile of sodium intake (601.8% AI) compared to those in the lowest, had increased odds of incident CKD by 64% in the fully adjusted model. Regarding the risk of incident CKD, a decreasing linear trend for potassium and magnesium and an increasing linear trend for sodium were noted (*p* for trend < 0.05, Table 3).

## 4. Discussion

In this prospective population-based study with a 3.6 years of follow-up, we found that higher intakes of magnesium and potassium and vitamins C, E, D, B12, and folate decreased the risk of incident CKD by 50% to 60%. However, sodium intake increased the risk of CKD by almost 60%.

Studies on the association between micronutrient intakes and CKD are limited [10,11,12]. Our findings regarding the inverse association between some dietary micronutrients and incident CKD are consistent with those of a cross-sectional study conducted in Australia [10], in which Strippoli *et al.* report that the intake of magnesium and folate based on EAR decreased the risk of CKD by 40%–45%. However, our findings showed 50%–60% reduction in risk of incident CKD with intakes 1 to 3-fold those of EAR. In contrast to Strippoli *et al.*, we found no significant association of phosphorus and calcium intake with CKD. Recently, the National Health and Nutrition Examination Survey (NHANES) showed that dairy foods with or without inorganic phosphates added, as well as cereals and grains with inorganic phosphates added, had a positive effect on serum phosphorus [12], a finding supported by the fact that higher serum phosphorus level increases kidney failure and mortality among patients with CKD [22]. Actually; although, the phosphorous serum level in subjects with end stage CKD increased and could have detrimental effects on health of subjects; this does not necessarily mean that phosphorus can increase the risk of incident CKD in healthy subjects. Therefore, dietary phosphorus restriction is important in subjects suffering various degrees of renal failure, whereas this nutrient is not a priority in subjects free of CKD.

Conflicting results exist on the association of sodium intake and CKD [11,23,24]. Our study emphasizes the remarkable role played by high dietary sodium in increasing risk of incident CKD by 64%. Data shows that chronic sodium overloads can increase the risk of incident CKD via adverse effects on renal function, proteinuria, decline in creatinine clearance, and GFR [23,24]. However, in a cross-sectional study by Sharma *et al.*, high dietary intake of sodium was associated with lower odds of CKD [11]. The proposed mechanisms by which low intake of sodium related to development or progression of CKD are activation of the sympathetic nervous and renin-angiotensin-aldosterone system, and LDL-C increase. However, more studies are required to confirm this association.

Potassium, another important nutrient, which is emphasized in dietary guidelines, was associated with incident CKD in our study. The potassium intake suggested by dietary guidelines is 4700 mg/day [25]. In the current study, reduced risk of incident CKD has been observed in dietary intakes of 5820 mg/day, indicating that higher intakes of potassium through fruits and vegetables should be encouraged. Also, in our study, the protective effect of magnesium has been shown, as magnesium intakes >EAR (581 mg) decreased risk of CKD by 60%, results indicating the importance of intakes of main food sources of magnesium including seeds, nuts, legumes, whole grains, and dark green vegetables in the prevention or controlling of several chronic diseases such as CKD. Our findings on the beneficial effects of vitamins C, E, D, and B12 on incident CKD have not been reported previously.

From a holistic point of view, encouraging the high intakes of vitamins C, D, E, B12, and folate, potassium, and magnesium and low intake of sodium, predominantly found in fruits, vegetables, dairy foods, whole grains, legumes, nuts, and fish can decrease the risk of incidence CKD. This point has been reported by studies indicating that micronutrient-rich dietary patterns lead to promoting the kidney function, thereby decreasing risk of renal failure [7,8,26]. Huang *et al.* have indicated that adherence to the Mediterranean diet, which emphasizes high intake of vegetables, legumes, fruits, nuts, cereals and is highly rich in vitamins, potassium, magnesium, calcium, and phosphorus, is associated with lower risk of CKD [7]. Similarly, other micronutrient-rich dietary patterns emphasizing sodium restriction, such as the DASH-style diet, with a high source of potassium and magnesium, increased eGFR, the effect of which might be explained by the known effects of this dietary pattern and its components on several cardio-metabolic risk factors, including improved plasma lipid profiles, blood pressure, insulin sensitivity, oxidative stress, inflammation, and endothelial dysfunction [8]. In contrast, high intakes of sodium and animal protein in a dietary pattern similar to those of Western lifestyles, representing a micronutrient-poor diet high in sodium, lead to high net endogenous acid production and increasing work load for the kidney. They also induce dyslipidemia, oxidative stress, inflammation, and disturbances of corticosteroid regulation, ultimately increasing the risk of incident CKD [9,26].

Several investigations have indicated that micronutrient intakes can play an important role in risk of CKD through modifying effects on hypertension, obesity, and diabetes [27,28,29]. It has been indicated that the high intake of folate, thiamin, riboflavin [27], vitamins A and C, B-carotene, total and non-heme iron, magnesium, calcium, phosphorus [28], and antioxidant micronutrients [29] are associated with decreased risk of incident hypertension, obesity, and diabetes. Regarding the adjustment of confounding effect of hypertension, obesity, and diabetes on risk of CKD in our study, it is probable that these micronutrients play important roles in reducing risk of CKD through other mechanisms, which unfortunately have yet to be fully elucidated. Since chronic inflammation is involved in the development of CKD [30], it seems reasonable that micronutrients by reducing inflammatory factors such as interleukin-6 (IL-6), total homocysteine, fibrinogen, and C-reactive protein (CRP) [31] play a protective role in risk of incident CKD. In a rat model of CKD, vitamins E and C decreased kidney damage by reducing renal superoxide production, arterial pressure, aortic superoxide production, renal inflammation, thereby preventing GFR and renal plasma flow reductions [32]. Also, some micronutrients including folate, B6, and B12 with antioxidant properties, by reducing oxidative damage and preventing DNA damage, are reported to have favorable effects on prevention of end-stage kidney diseases [33].

Some strengths of the current study deserve to be mentioned. First, to our knowledge, this is the first study investigating the association between micronutrient intake and risk of incident CKD in a population-based cohort study. Furthermore, the population-based cohort subjects whose socio-demographic information had been collected in detail facilitated a thorough investigation of possible confounders. Also, using a FFQ specially developed and validated in our population are strengths of our study. However, our study does have some limitations. First, data on food additives and dietary supplements were not available. Second, in the current study, we defined chronic kidney diseases, as in most epidemiologic studies, based on a limited number of isolated creatinine measurements that were not repeated within three months to confirm a chronic reduction in GFR. Third, despite adjusting for the confounding effects of various variables in our analysis, residual confounding due to unknown or unmeasured confounders—including socioeconomic factors such as household income, occupation status, and fluid intakes—cannot be excluded.

## 5. Conclusions

In conclusion, the results of our investigation suggest that higher intakes of several micronutrients such as vitamins C, E, D, B12, folate, magnesium, and potassium decrease the risk of CKD, whereas high intakes of sodium are associated with increased risk of incident CKD, findings emphasizing that dietary sources of renal-protective nutrients should be encouraged among the general population.

## Figures and Tables

**Table 1:** Demographic and clinical characteristics of chronic kidney disease (CKD) and non-CKD subjects at baseline in the Tehran Lipid and Glucose Study.

	All (*n* = 1692)	Non-CKD (*n* = 1519)	CKD (*n* = 173)	*p*
Age (years)	43.3 ± 11.4	41.4 ± 10.6	51.2 ± 11.4	<0.001
Men (%)	49.2	53.0	31.0	<0.001
Smoking (%)	12.0	12.6	8.1	<0.001
Light physical activity (%)	60.6	58.6	78.0	<0.001
Energy intake (kcal)	2285 ± 18	2288 ± 20	2270 ± 43	0.686
BMI (kg/m^2^)	27.6 ± 0.1	27.5 ± 0.1	28.2 ± 0.2	0.016
SBP (mmHg)	113.0 ± 0.4	111.9 ± 0.8	113.2 ± 0.7	0.125
DBP (mmHg)	74.4 ± 0.2	74.4 ± 0.2	74.4 ± 0.6	0.993
Hypertension (%)	23.2	21.1	31.6	<0.001
Diabetes (%)	7.2	6.1	16.1	<0.001
Triglycerides (mL/dL)	130.0 (83.0–171.0)	127.0 (81.0–166.0)	146.0 (94.0–189.5)	<0.001
Cholesterol (mL/dL)	190.8 ± 0.8	189.9 ± 0.9	194.5 ± 2.0	0.045
LDL-C (mL/dL)	119.1 ± 0.7	118.8 ± 0.8	120.5 ± 1.8	0.043
HDL-C (mL/dL)	41.9 ± 0.2	43.5 ± 0.2	41.4 ± 0.5	0.003
FPG (mL/dL)	93.3 ± 0.5	93.1 ± 0.6	93.8 ± 1.3	0.694

BMI: body mass index; SBP: systolic blood pressure; DBP: diastolic blood pressure; LDL-C: low density lipoprotein cholesterol; HDL-C: low density lipoprotein cholesterol; FPG: fasting plasma glucose. Data are mean ± SE or median (25–75 interquartile range), unless otherwise stated. All variables were adjusted for age except categorical ones. Chronic kidney disease was defined as estimated glomerular filtration rate <60 mL/min/1.73 m^2^.

**Table 2:** Odds ratio (95% confidence interval) for incident chronic kidney disease according to quintiles of vitamin intakes.

	Quintiles			*p* for Trend *
	Q 1	Q 3	Q 5	
Thiamin (mg) (% EAR)	1.11(119.2)	1.82 (192.0)	2.99 (332.7)	
Model 1	Ref.	0.97 (0.67–1.40)	0.88 (0.61–1.29)	0.212
Model 2	Ref.	1.20 (0.75–1.92)	1.11 (0.57–2.16)	0.823
Riboflavin (mg) (% EAR)	1.04 (106.5)	1.88 (191.2)	3.29 (329.3)	
Model 1	Ref.	1.24 (0.84–1.83)	1.43 (0.98–2.10)	0.249
Model 2	Ref.	1.35 (0.84–2.16)	1.70 (0.92–3.14)	0.396
Niacin (mg) (% EAR)	12.52 (110.0)	20.82 (181.7)	34.41 (315.5)	
Model 1	Ref.	1.08 (0.73–1.60)	1.14 (0.78–1.68)	0.997
Model 2	Ref.	1.26 (0.78–2.04)	1.56 (0.80–3.07)	0.336
Pyridoxine (mg) (% EAR)	1.11 (96.6)	1.08 (160.2)	3.08 (265.8)	
Model 1	Ref.	1.25 (0.85–1.85)	1.37 (0.94–2.01)	0.242
Model 2	Ref.	1.37 (0.85–2.2)	1.60 (0.83–3.09)	0.357
Folate (µg) (% EAR)	245.8 (104.9)	448.2 (138.8)	628.2 (258.8)	
Model 1	Ref.	0.87 (0.60–1.25)	0.62 (0.42–0.92)	0.027
Model 2	Ref.	0.78 (0.50–1.23)	0.44 (0.24–0.80)	0.007
Cobalamin (µg) (% EAR)	2.2 (109.1)	3.6 (181.8)	7.4 (369.7)	
Model 1	Ref.	0.72 (0.50–1.05)	0.58 (0.40–0.86)	0.005
Model 2	Ref.	0.71 (0.46–1.09)	0.57 (0.34–0.93)	0.023
Vitamin C (mg) (% EAR)	52.2 (85.6)	124.9 (187.0)	268.2 (445.4)	
Model 1	Ref.	0.78 (0.48–1.25)	0.70 (0.43–1.15)	0.077
Model 2	Ref.	0.62 (0.36–1.05)	0.38 (0.21–0.69)	<0.001
Vitamin A (µg) (% EAR)	197.4 (35.2)	425.8 (77.0)	859.9 (188.2)	
Model 1	Ref.	1.04 (0.60–1.79)	1.46 (0.88–2.44)	0.377
Model 2	Ref.	0.85 (0.46–1.56)	1.15 (0.61–2.16)	0.837
Vitamin D (µg) (% EAR)	0.61 (6.1)	1.18 (15.3)	4.09 (40.9)	
Model 1	Ref.	0.59 (0.36–0.95)	0.35 (0.20–0.62)	<0.001
Model 2	Ref.	0.64 (0.38–1.07)	0.39 (0.21–0.70)	0.002
Vitamin E (mg) (% EAR)	6.06 (50.5)	10.76 (89.7)	17.61 (163.3)	
Model 1	Ref.	0.79 (0.47–1.31)	0.72 (0.43–1.21)	0.079
Model 2	Ref.	0.67 (0.38–1.19)	0.45 (0.22–0.92)	0.005

EAR: Estimated Average Requirement; Model 1: Adjusted for age; Model 2: Further adjusted for sex, and energy intake, serum triglycerides, serum cholesterol, BMI, hypertension, diabetes, physical activity, and smoking; * Based on logistic regression model using median intake of vitamins in each quintile as a continuous variable.

**Table 3:** Odds ratio (95% confidence interval) for incident chronic kidney disease according to quintiles of mineral intakes.

	Quintiles			*p* for Trend *
	Q 1	Q 3	Q 5	
Sodium (g) (% AI)	1.85 (123.0)	3.52 (234.8)	7.87 (601.8)	
Model 1	Ref.	1.18 (0.79–1.76)	1.56 (1.06–2.30)	0.042
Model 2	Ref.	1.14 (0.72–1.83)	1.64 (1.03–2.61)	0.041
Potassium (g) (% AI)	2.15 (45.8)	3.59 (75.1)	5.82 (131.6)	
Model 1	Ref.	0.76 (0.46–1.25)	0.79 (0.49–1.30)	0.093
Model 2	Ref.	0.71 (0.40–1.26)	0.47 (0.23–0.97)	0.039
Calcium (mg) (% EAR)	619.9 (84.4)	931.7 (146.3)	1660.2 (249.8)	
Model 1	Ref.	0.89 (0.53–1.45)	1.12 (0.69–1.83)	0.364
Model 2	Ref.	0.81 (0.45–1.46)	0.79 (0.39–1.57)	0.444
Magnesium (mg) (% EAR)	224.9 (77.9)	356.2 (118.3)	581.3 (195.7)	
Model 1	Ref.	0.88 (0.61–1.29)	0.70 (0.47–1.03)	0.046
Model 2	Ref.	0.69 (0.43–1.09)	0.41 (0.22–0.76)	0.002
Phosphorus (mg) (% EAR)	820.4 (141.4)	1392.6 (239.9)	2206.5 (401.1)	
Model 1	Ref.	0.96 (0.66–1.39)	0.90 (0.62–1.32)	0.323
Model 2	Ref.	0.73 (0.40–1.33)	0.77 (0.48–1.24)	0.187
Selenium (µg) (% EAR)	60.0 (133.5)	102.4 (228.7)	175.1 (430.4)	
Model 1	Ref.	0.93 (0.64–1.36)	0.87 (0.59–1.26)	0.926
Model 2	Ref.	1.10 (0.70–1.74)	1.13 (0.62–2.04)	0.869
Zinc (mg) (% EAR)	6.5 (84.8)	10.7 (136.6)	17.4 (223.7)	
Model 1	Ref.	0.96 (0.65–1.43)	1.15 (0.78–1.69)	0.992
Model 2	Ref.	0.97 (0.59–1.59)	1.29 (0.65–2.58)	0.959

AI: Adequate Intake; EAR: Estimated Average Requirement; Model 1: Adjusted for age; Model 2: Further adjusted for sex, and energy intake, serum triglycerides, serum cholesterol, BMI, hypertension, diabetes, physical activity, and smoking; * Based on logistic regression model using median intake of minerals in each quintile as a continuous variable.
